# The Potential Role of Serum Tissue Inhibitor of Metalloproteinase-2 (TIMP-2) as a Biomarker of Fibrosis in Patients With Metabolic Dysfunction-Associated Fatty Liver Disease (MAFLD)

**DOI:** 10.7759/cureus.96047

**Published:** 2025-11-03

**Authors:** Aalia Tahseen, Suchitra Kumari, Manaswini Mangaraj, Manas K Panigrahi

**Affiliations:** 1 Biochemistry, All India Institute of Medical Sciences (AIIMS) Bhubaneswar, Bhubaneswar, IND; 2 Gastroenterology, All India Institute of Medical Sciences (AIIMS) Bhubaneswar, Bhubaneswar, IND

**Keywords:** alt, biomarkers, fibroscan, fibrosis, ggt, hepatic stellate cells, liver injury, metabolic-associated fatty liver disease (mafld), metabolic syndrome, timp-2

## Abstract

Background: Activation of hepatic stellate cells (HSCs) in response to liver injury increases extracellular matrix (ECM) deposition and upregulation of tissue inhibitor of metalloproteinase-2 (TIMP-2), promoting fibrogenesis, a key feature of metabolic dysfunction-associated fatty liver disease (MAFLD). This study was conducted to determine the diagnostic potential of serum TIMP-2 levels in patients with MAFLD/nonalcoholic fatty liver disease (NAFLD).

Methods: This case-control study included 100 MAFLD patients and 100 healthy controls. Demographic profiling, ultrasonography, and FibroScan with controlled attenuation parameter (CAP) scoring were conducted. Serum TIMP-2 was quantified using enzyme-linked immunosorbent assay (ELISA), and routine biochemical parameters (liver function test, kidney function test, fasting plasma glucose, postprandial blood sugar, HbA1c, total cholesterol, triglycerides, and GGT) were assessed using fully automated chemistry analyzers. Receiver operating characteristic (ROC) curve analysis was performed to assess the diagnostic utility of TIMP-2.

Results: Serum TIMP-2 levels were significantly higher in MAFLD patients (p < 0.001) compared with controls. Serum TIMP-2 showed a significant positive correlation with ALT (rho = 0.569, p < 0.001), GGT (rho = 0.551, p < 0.001), fasting plasma insulin (rho = 0.562, p < 0.001), and FibroScan scores (r = 0.560, p < 0.001). ROC analysis yielded an AUC of 0.933 (95% CI: 0.889-0.963) with a TIMP-2 cut-off value of 8.909, showing a sensitivity of 91% and specificity of 99%.

Conclusion: Serum TIMP-2 levels are significantly elevated in MAFLD and show strong correlations with liver enzymes and CAP scores, suggesting a relationship with hepatic fat accumulation and liver injury. Additionally, associations with lipid levels, white blood cell count, and neutrophils imply a link to systemic inflammation and liver fibrosis, highlighting the promising role of TIMP-2 as a noninvasive, blood-based biomarker for early detection and monitoring of liver fibrosis. Further studies are needed to substantiate the diagnostic potential of serum TIMP-2 in MAFLD patients.

## Introduction

Metabolic dysfunction-associated fatty liver disease (MAFLD), previously known as nonalcoholic fatty liver disease (NAFLD), is defined as hepatic steatosis in the absence of significant alcohol intake, occurring with overweight or obesity, type 2 diabetes, or other metabolic dysregulation, irrespective of other liver disease etiologies [1]. It is primarily associated with metabolic risk factors such as obesity, insulin resistance, and dyslipidemia. MAFLD encompasses a spectrum of liver conditions, including simple fatty liver, nonalcoholic steatohepatitis (NASH), advanced liver fibrosis, cirrhosis, and hepatocellular carcinoma (HCC).

The global incidence of MAFLD has been rising, driven largely by lifestyle changes and the increasing prevalence of noncommunicable diseases. In Western countries, MAFLD affects approximately 32% of adults, while in India, its prevalence ranges from 6.7% to 55.1%. In the Odisha region specifically, the prevalence is reported to be 10% to 33%. The escalating rates of MAFLD have resulted in considerable economic and healthcare burdens [2,3].

MAFLD is associated with several histological changes in the liver, such as steatosis, lobular inflammation, hepatocellular ballooning, and fibrosis. These changes stem from complex alterations in hepatic structure involving not only hepatocytes but also ongoing remodeling of the extracellular matrix (ECM). Liver fibrosis, a common progression of MAFLD, is regarded as a wound-healing response resulting from an imbalance between the synthesis and degradation of ECM components. Matrix metalloproteinases (MMPs), a family of zinc-dependent enzymes, play an essential role in ECM degradation [1,4]. The balance between MMPs and their endogenous tissue inhibitors (TIMPs) is crucial for regulating ECM turnover. When this balance is disrupted, excessive collagen production and abnormal ECM deposition occur, resulting in fibrosis that may eventually progress to cirrhosis or hepatocellular carcinoma [1,4].

TIMP-2, located on chromosome 17q25, specifically regulates the activity of MMP-2 and plays a critical role in cellular processes such as development, apoptosis, and maintenance of tissue integrity [4,5]. In response to liver injury, activated hepatic stellate cells (HSCs) increase the synthesis of ECM components and produce more TIMP, which inhibits MMPs and reduces matrix degradation. This accumulation of collagen and formation of cross-links in cirrhosis leads to increased tissue stiffness, fibrosis, and irreversible liver damage. FibroScan is a noninvasive diagnostic tool widely used to assess liver stiffness, which correlates with the degree of fibrosis. This technique, based on transient elastography, allows accurate measurement of liver stiffness and has become an essential method for evaluating fibrosis. Compared with liver biopsy, it is safer, quicker, and eliminates the risks associated with invasive procedures [6,7].

MMPs have been shown to positively correlate with elevated levels of alanine aminotransferase (ALT) and gamma-glutamyl transferase (GGT), with GGT serving as a more sensitive marker of oxidative stress and disease progression, while ALT remains relatively specific for liver injury. Although previous studies have reported correlations between MMPs and these enzymes in patients with MAFLD, they are not diagnostic markers. In this study, we evaluated the relationship between TIMP-2, ALT, and GGT to explore their potential as indicators of liver injury and oxidative stress. As endogenous inhibitors of MMPs, TIMPs, particularly TIMP-2, may serve as biomarkers for hepatic damage and fibrosis. While ALT and GGT are not specific for diagnosing MAFLD, their correlation with TIMP-2 provides insight into its pathophysiological relevance rather than diagnostic utility.

Currently, no pharmacological treatments are approved for MAFLD, underscoring the need to identify key molecular targets in disease progression. TIMP-2 has emerged as a promising candidate due to its role in regulating MMP activity [1,8,9]. Current diagnostic techniques, such as liver biopsy, are invasive and carry associated risks. As an endogenous inhibitor of MMPs involved in matrix degradation, TIMP-2 has been proposed as a promising biomarker for MAFLD. Elevated levels of circulating TIMP-2 have been observed in individuals with MAFLD. Given the complexity and increasing global prevalence of MAFLD, understanding the role of molecules like TIMP-2 in its pathogenesis, as well as their potential as therapeutic targets and biomarkers, is critical for advancing effective treatment strategies.

This study was conducted to quantify serum TIMP-2 levels in patients with MAFLD and to evaluate their association with key markers of liver fibrosis, including ALT, GGT, and FibroScan results.

This article was previously presented as an oral paper at the 49th ACBICON (Association of Clinical Biochemists of India) held in Kerala from September 13 to 16, 2023.

## Materials and methods

Subjects

A case-control study was conducted in the Department of Biochemistry at the All India Institute of Medical Sciences (AIIMS), Bhubaneswar, Odisha, India. The study included 100 adult patients (aged >18 years) attending the Medical Gastroenterology Outpatient Department who were clinically diagnosed with MAFLD based on medical history, clinical presentation, biochemical parameters, and radiological findings (USG and FibroScan). These individuals were enrolled as cases. An equal number of age- and sex-matched individuals without evidence of fatty liver on imaging were recruited as controls. Patients with a history or laboratory evidence of hepatitis B or C infection, autoimmune hepatitis, chronic alcohol consumption, Wilson’s disease, alpha-1 antitrypsin deficiency, or hemochromatosis were excluded from the study.

Sample size

Sample size was calculated using the following formula [10]:

$n = \frac{(Z_{&alpha;} \sqrt{2\bar{p}\bar{q}} + Zᵦ\sqrt{p_{1}q_{1}} + p_{0}q_{0})^{2}}{(p_{1}-p_{0})^{2}}$

where

$p_{1}= \frac{p_{0}&times;R}{1+ p_{0} (R-1)}$

$\bar{p}=&frac12; (p_{1}+p_{0})$

$\bar{q}= 1- \bar{p}$

$q_{1}= 1-p_{1}$

$q_{0}=1-p_{0}$

where R is the hypothesized relative risk associated with exposure, p_0_ is the relative frequency of exposure among controls, the value of Zα is 1.96 for α = 0.05, and the value of Zᵦ is 0.84 for power = 90%.

The calculated sample size was 100 cases and 100 controls.

Methods

This study was approved by the Institutional Ethics Committee of AIIMS Bhubaneswar (IEC/AIIMS BBSR/PG Thesis/2021-22/54). Following ethical approval, the research work commenced in August 2021 and was completed in September 2023. A total of 100 cases and 100 controls were evaluated using a structured questionnaire, biochemical investigations, clinical examination, abdominal ultrasonography, and FibroScan findings (CAP scores) [11].

Clinical evaluation

A structured, self-designed questionnaire was used to collect comprehensive clinical and lifestyle data, including information on the duration and family history of MAFLD, previous alcohol consumption, smoking status, coexisting medical conditions, and current medications (see the Appendices). Blood pressure was recorded using standardized measurement protocols. Anthropometric evaluations, including measurements of height, weight, waist circumference, and hip circumference, were conducted to calculate body mass index (BMI) and assess central obesity. The extent of hepatic steatosis was quantified using the controlled attenuation parameter (CAP), derived from FibroScan, a noninvasive elastography technique.

Biochemical analysis

Following written informed consent, 3 mL of venous blood was collected from each participant via venipuncture for biochemical analysis. The blood samples were processed within one hour to separate serum, which was then stored at −80 °C until further use. Serum levels of tissue inhibitor of metalloproteinase-2 (TIMP-2) were measured using a commercially available human TIMP-2 enzyme-linked immunosorbent assay (ELISA) kit (Elabscience, Texas), following the manufacturer’s instructions.

Briefly, standards and samples were added to designated wells of the ELISA plate and incubated. After washing, the biotinylated detection antibody working solution was added and incubated, followed by additional washing steps and the addition of HRP-conjugated working solution. After incubation, the substrate solution was added, and the reaction was terminated with the stop solution. Absorbance was read at 450 ± 2 nm using an ELISA reader (BioTek ELx50, Vermont).

Routine biochemical parameters, including liver function tests (total bilirubin, aspartate aminotransferase (AST), alanine aminotransferase (ALT), alkaline phosphatase (ALP), total protein, and albumin), kidney function tests (urea, creatinine, uric acid, sodium, potassium, and chloride), fasting and postprandial plasma glucose, HbA1c, lipid profile (total cholesterol, HDL-C, LDL-C, and triglycerides), and gamma-glutamyl transferase (GGT), were analyzed using a fully automated chemistry analyzer (Beckman Coulter AU5800 platform). All biochemical results, along with clinical and anthropometric data, were systematically recorded in individual case report forms.

Statistical analysis

Data obtained from the questionnaire, along with biochemical and clinical evaluations, were statistically analyzed using IBM SPSS Statistics for Windows, Version 21 (Released 2012; IBM Corp., Armonk, New York). Descriptive statistics, including frequencies and percentages, were used to summarize the demographic characteristics of both patients and controls. Continuous variables were expressed as means with standard deviations (SD).

The chi-square (χ²) test was used to assess potential differences in genotype distributions between patients and controls. Unconditional logistic regression models were used to evaluate the association between TIMP-2 polymorphisms and the risk of MAFLD, with odds ratios (ORs) and 95% confidence intervals (CIs) calculated. ROC curve analysis was performed to assess the sensitivity and specificity of the biomarkers, and the area under the ROC curve (AUC) was computed to evaluate their diagnostic accuracy.

Among participants classified as MAFLD-positive, serum TIMP-2 levels were further correlated with fasting blood sugar, HbA1c, mean corpuscular volume (MCV), mean corpuscular hemoglobin (MCH), red cell distribution width (RDW), total leukocyte count (TLC), monocytes, total bilirubin (T.BIL), indirect bilirubin (I.BIL), AST, ALP, total protein, albumin, creatinine, sodium, potassium, chloride, total cholesterol, triglycerides, HDL, LDL, and established markers of liver fibrosis, i.e., alanine aminotransferase (ALT), gamma-glutamyl transferase (GGT), and FibroScan CAP values using Pearson’s correlation. Statistical significance was defined as a p-value ≤ 0.05.

## Results

The clinicodemographic characteristics of the study participants are summarized in Table 1. There were no statistically significant differences between the MAFLD and control groups with respect to age or sex distribution (p > 0.05). In contrast, significant differences were observed between the two groups in terms of body weight, body mass index (BMI), waist and hip circumferences, family history of metabolic conditions, smoking status, and the prevalence of hypertension and diabetes (p < 0.001). The mean BMI of participants with MAFLD (30 ± 2.5 kg/m²) was significantly higher than that of the control group (24.8 ± 0.7 kg/m²). Notably, all individuals in both groups were nonalcoholic.

**Table 1:** Clinicodemographic profile of study participants Statistical significance (p-value) was calculated using the following: ^#^: Data expressed as mean ± SD; *: Data expressed as count (%); ^χ^: Chi-square test; t: Independent sample t-test; –: Chi-square test not performed because of null cells. p < 0.05 was considered statistically significant.

Parameters^#^	Category	Controls (N=100)	Cases (N=100)	p-value^t^
Age (years)		50.03 ± 10.52	51.98 ± 10.52	0.187
Gender^*^	Male	79 (79)	78 (78)	0.863^χ^
	Female	21 (21)	22 (22)	
Height (cm)		155.6 ± 3.8	156.7 ± 4.5	0.074
Weight (kg)		60.1 ± 2.7	74.4 ± 6.9	<0.001
BMI (kg/m^2^)		24.8 ± 0.7	30 ± 2.5	<0.001
Waist circumference (cm)		76.6 ± 3.7	85.2 ± 8.3	<0.001
Hip circumference (cm)		80.8 ± 4.1	85.2 ± 6.6	<0.001
Smoker^*^	No	100 (100)	88 (88)	<0.001^χ^
	Yes	0 (0)	12 (12)	
Alcoholic^*^	No	100 (100)	100 (100)	-
	Yes	0 (0)	0 (0)	
Hypertensive^*^	No	100 (100)	85 (85)	<0.001^χ^
	Yes	0 (0)	15 (15)	
Diabetic^*^	No	100 (100)	80 (80)	<0.001^χ^
	Yes	0 (0)	20 (20)	
Family history^*^	No	100 (100)	98 (98)	0.155^χ^
	Yes	0 (0)	2 (2)	

Biochemical analysis presented in Table 2 showed significantly elevated fasting insulin, fasting blood sugar (FBS), and HbA1c levels in the MAFLD group compared to controls (p < 0.001). Liver function tests revealed significantly higher ALT and GGT levels in the MAFLD group (76 U/L and 42.8 U/L, respectively) compared to controls (32 U/L and 22.3 U/L) (p < 0.001). Other parameters, including bilirubin, AST, ALP, TP, and albumin, were also significantly higher in the MAFLD group (p < 0.005). Lipid profile analysis showed increased total cholesterol, triglycerides, and LDL in the MAFLD group (p < 0.001).

**Table 2:** Biochemical parameters of the study population Statistical significance (p value) was calculated using the following: ^#^: Data expressed as mean ± SD; *: Data expressed as count (%); t: Independent sample t-test. p < 0.05 was considered statistically significant. HbA1c, glycated hemoglobin; RBC, red blood cell count; PCV, packed cell volume; MCV, mean corpuscular volume; MCH, mean corpuscular hemoglobin; MCHC, mean corpuscular hemoglobin concentration; RDW, red blood cell distribution width; TLC, total leucocyte count; AST, aspartate transferase; ALT, alanine transaminase; ALP, alkaline phosphatase; GGT, gamma glutamyl transferase; Na, sodium; K, potassium; Cl, chloride; TG, triglycerides; HDL, high-density lipoprotein; LDL, low-density lipoprotein.

Parameters ^#^	Controls (N = 100)	Cases (N = 100)	p-value ^t^
Fasting blood sugar (mg/dL)	80.8 ± 7	102.2 ± 20.1	<0.001
HbA1c (in %)	5 ± 0.6	5.7 ± 1	<0.001
RBC count (million cells/mm^3^)	4.5 ± 0.7	4.8 ± 0.7	0.002
PCV (%)	41.4 ± 3.2	40 ± 4.5	0.011
MCV (fL)	86.2 ± 5.2	82.1 ± 7	<0.001
MCH (pg)	29.8 ± 1.9	27.2 ± 3.2	<0.001
MCHC (g/dL)	32.7 ± 1.1	32.2 ± 1.4	0.007
RDW (%)	12.6 ± 1.4	13.9 ± 1.7	<0.001
Total leucocyte count (1000 cells/mm^3^)	6.9 ± 1.9	8.1 ± 1.9	<0.001
Neutrophils (% of TLC)	60 ± 11.8	63.2 ± 9.8	0.037
Monocytes (% of TLC)	1.6 ± 1.2	3.3 ± 1.7	<0.001
Total bilirubin (mg/dL)	0.6 ± 0.3	0.8 ± 0.6	<0.001
Direct bilirubin (mg/dL)	0.1 ± 0.1	0.2 ± 0.2	0.047
Indirect bilirubin (mg/dL)	0.4 ± 0.2	0.7 ± 0.6	<0.001
AST (U/L)	25 ± 9.2	50.7 ± 23.8	<0.001
ALT (U/L)	32 ± 9.3	76 ± 54.8	<0.001
ALP (U/L)	72.9 ± 14	99.7 ± 26.8	<0.001
Total protein (g/dL)	6.8 ± 0.7	7.3 ± 0.7	<0.001
Albumin (g/dL)	3.9 ± 0.6	4.2 ± 0.6	<0.001
Globulin (g/dL)	2.9 ± 0.7	3.1 ± 0.5	0.018
GGT (U/L)	22.3 ± 7.7	42.8 ± 16.7	<0.001
Creatinine (mg/dL)	0.7 ± 0.3	1 ± 0.3	<0.001
Uric acid (mg/dL)	5.3 ± 1.2	6.2 ± 2	<0.001
Na (mEq/L)	134.8 ± 3	137.6 ± 4	<0.001
K (mEq/L)	3.7 ± 0.5	4.1 ± 0.5	<0.001
Cl (mmol/L)	98 ± 5.6	101.1 ± 4.9	<0.001
Fasting plasma insulin (mIU/L)	5.1 ± 1.2	10.6 ± 4.5	<0.001
Total cholesterol (mg/dL)	145.3 ± 19.3	203.9 ± 44.7	<0.001
TG (mg/dL)	68.4 ± 17.3	168.7 ± 88.9	<0.001
HDL (mg/dL)	39.7 ± 9.2	30.5 ± 4.8	<0.001
LDL (mg/dL)	100.5 ± 21.1	133.8 ±40.7	<0.001

FibroScan analysis comparing grade I fatty liver among the study groups showed significantly higher values in the MAFLD group compared to the control group (p < 0.001) (Table 3). Additionally, serum TIMP-2 levels were significantly elevated in the MAFLD group (9.2 ng/mL) compared to the control group (8.5 ng/mL) (p < 0.001) (Table 3).

**Table 3:** Comparison of serum TIMP-2 and FibroScan levels in the study group Statistical significance (P value) was calculated using the following: ^#^: Data expressed as mean ± SD; t: Independent sample t-test. P < 0.05 was considered statistically significant. CAP, controlled attenuation parameter; TIMP-2, tissue inhibitor of metalloproteinase 2.

Parameters^#^	Controls (N = 100)	Cases (N = 100)	p-value^t^
FibroScan (in CAP db/m) (Grade I fatty liver)	31.7 ± 9.8	297.9 ± 33.8	<0.001
Serum TIMP-2 levels (ng/mL)	8.5 ± 0.5	9.2 ± 0.6	<0.001

**Figure 1:**
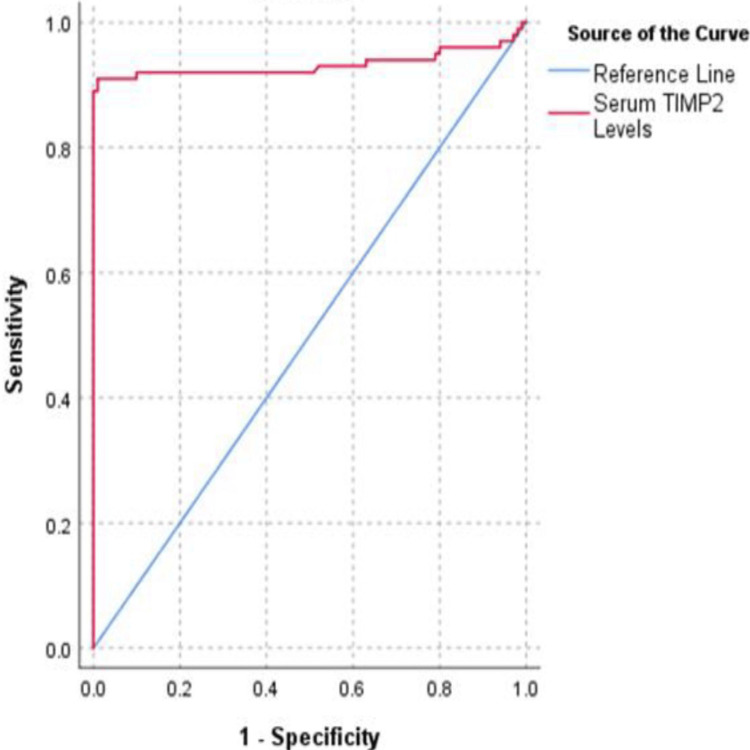
ROC curve analysis for serum TIMP-2 levels in the diagnosis of MAFLD The figure illustrates the ROC analysis for serum TIMP-2 levels in diagnosing MAFLD. The AUC for serum TIMP-2 was 0.933 (95% CI, 0.889–0.963; p < 0.001). Based on the Youden Index of 0.900, the optimal cutoff value was determined to be 8.909 ng/mL, with values above this threshold being suggestive of MAFLD. At this cutoff, the test demonstrated a sensitivity of 91% and a specificity of 99%. The corresponding LR+ and LR– were 91 and 0.09, respectively, indicating that serum TIMP-2 is a significantly conclusive diagnostic marker for MAFLD. ROC, receiver operating characteristic; LR+, positive likelihood ratio; LR–, negative likelihood ratio; AUC, area under the curve; MAFLD, metabolic dysfunction–associated fatty liver disease.

To assess the diagnostic value of serum TIMP-2, ROC analysis was performed, yielding an area under the curve (AUC) of 0.933 (95% CI: 0.889-0.963, p < 0.001), as shown in Figure 1. The optimal cutoff value for serum TIMP-2, based on the Youden index, was determined to be 8.909 ng/mL. The likelihood ratio for a positive test (LR+) was 91, while the likelihood ratio for a negative test (LR−) was 0.09.

**Figure 2:**
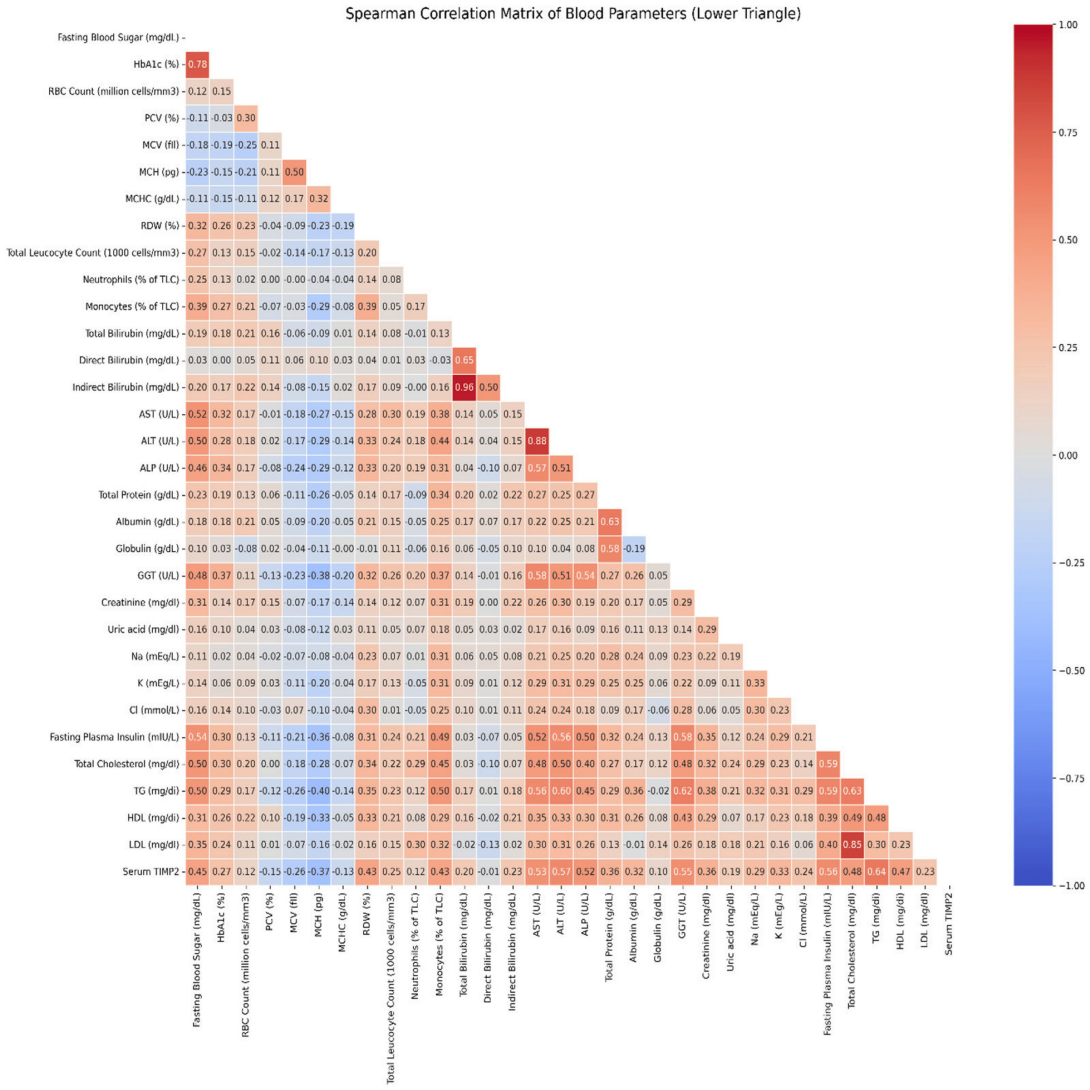
Correlation heat map of spearman correlation coefficients of serum TIMP-2 levels with biomarkers and FibroScan levels

Serum TIMP-2 levels showed significant associations with various markers of metabolic dysfunction, suggesting its potential utility as a noninvasive biomarker for MAFLD. Positive correlations with fasting glucose, HbA1c, and insulin point to its involvement in insulin resistance. Strong associations with liver enzymes, bilirubin levels, and FibroScan CAP scores indicate a relationship with hepatic steatosis and liver injury. Additionally, correlations with lipid parameters and inflammatory markers support its role in the metabolic syndrome spectrum. Inverse associations with red blood cell indices and positive correlations with renal markers and electrolytes reflect broader systemic metabolic disruption. Notably, the positive correlation between TIMP-2 and FibroScan values also suggests a potential link to liver fibrosis. Spearman correlation analysis (Table 4 and Figure 2), based on data from 200 participants, further validated these findings, underscoring the diagnostic relevance of TIMP-2 in metabolic dysfunction and MAFLD-related liver damage.

**Table 4:** Correlation between serum TIMP-2 and biochemical parameters among the study groups Correlation is significant at the 0.01 level (two-tailed). ρ (Rho): Spearman correlation coefficient. p < 0.05 was considered statistically significant. *: p < 0.05 was considered statistically significant. TIMP-2, tissue inhibitor of metalloproteinase 2; FBS, fasting blood sugar; HbA1c, glycated hemoglobin; PCV, packed cell volume; RBC, red blood cell count; MCV, mean corpuscular volume; MCH, mean corpuscular hemoglobin; RDW, red blood cell distribution width; TLC, total leucocyte count; T.BIL, total bilirubin; I.BIL, indirect bilirubin; AST, aspartate transferase; ALT, alanine transaminase; ALP, alkaline phosphatase; GGT, gamma glutamyl transferase; Na, sodium; K, potassium; Cl, chloride; TG, triglycerides; HDL, high-density lipoprotein; LDL, low-density lipoprotein.

Parameters	Category	Serum TIMP-2 (ng/mL)
FBS (mg/dL)	rho value	0.453
	p value	0.001*
HbA1c (%)	rho value	0.265
	p value	0.003
PCV (%)	rho value	-0.145
	p value	0.04*
MCV (fl)	rho value	-0.258
	p value	0.001*
MCH (pg)	rho value	-0.375
	p value	0.001*
RDW (%)	rho value	0.432
	p value	0.001*
Total leucocyte count (1000 cells/mm^3^)	rho value	0.254
	p value	0.001*
Monocytes (% of TLC)	rho value	0.431
	p value	0.001*
T.BIL (mg/dL)	rho value	0.201
	p value	0.004*
I.BIL (mg/dL)	rho value	0.226
	p value	0.001*
AST (U/L)	rho value	0.533
	p value	0.001*
ALT (U/L)	rho value	0.569
	p value	0.001*
ALP (U/L)	rho value	0.515
	p value	0.001*
GGT (U/L)	rho value	0.551
	p value	0.001*
Total protein (g/dL)	rho value	0.360
	p value	0.001*
Albumin (g/dL)	rho value	0.323
	p value	0.001*
Creatinine (mg/dL)	rho value	0.363
	p value	0.001*
Na (mEq/L)	rho value	0.294
	p value	0.001*
K (mEq/L)	rho value	0.333
	p value	0.001*
Cl (mmol/L)	rho value	0.244
	p value	0.001*
Fasting plasma insulin (mIU/L)	rho value	0.562
	p value	0.001*
Total cholesterol (mg/dL)	rho value	0.476
	p value	0.001*
TG (mg/dL)	rho value	0.643
	p value	0.001*
HDL (mg/dL)	rho value	0.468
	p value	0.001*
LDL (mg/dL)	rho value	0.235
	p value	0.001*
FibroScan (in CAP db/m) (Grade I fatty liver)	rho value	0.560
	p value	0.001*

Table 4 presents the Spearman correlation between serum TIMP-2 levels and a range of metabolic and biochemical variables, including glycemic markers (fasting blood sugar, HbA1c), red blood cell indices (MCV, MCH, RDW), white blood cell parameters (TLC, monocytes), liver function markers (T. BIL, I. BIL, AST, ALT, ALP, GGT), serum proteins (total protein, albumin), renal function indicators (creatinine, sodium, potassium, chloride), lipid profile components (total cholesterol, triglycerides, HDL, LDL), and FibroScan values. Data from 200 participants revealed strong positive correlations between TIMP-2 and these variables, emphasizing its potential role as a diagnostic marker in MAFLD and metabolic disturbances.

## Discussion

The present study was designed to quantitatively assess serum levels of TIMP-2 and explore its relationship with markers of hepatic injury, specifically serum ALT, GGT, and FibroScan values. This case-control study included 200 participants, divided into two groups: 100 individuals with metabolic dysfunction-associated fatty liver disease (MAFLD) and 100 healthy controls. MAFLD, characterized by fat accumulation in the liver without alcohol consumption, is increasingly prevalent worldwide. Previous studies have highlighted the higher incidence of MAFLD in middle-aged and elderly populations. In the present study, the average age of participants in the MAFLD group was 51.9 years, which aligns with findings by Frith et al., who categorized their subjects into three age groups and reported no significant age difference among them [12-14].

Although the male cohort in our study was larger than the female cohort, this gender disparity was not statistically significant, consistent with studies by Zhou et al. and Chen et al., where the male-to-female ratio in MAFLD patients was also comparable [13,15]. This pattern may reflect the protective effects of estrogen in premenopausal women, although after menopause, the incidence of MAFLD in women surpasses that in age-matched men. Loomis et al. demonstrated that BMI correlates directly with the risk of MAFLD, with an increased likelihood of MAFLD as BMI rises. Similarly, Lu et al. identified high BMI as a modifiable risk factor for MAFLD, a finding corroborated by the present study, which also noted a marked increase in BMI in the MAFLD group compared to controls [16].

In addition to BMI, we also assessed anthropometric measures such as height, body weight, waist circumference, and hip circumference, all of which were significantly higher in the MAFLD group compared to the control group. These results are consistent with studies by Zheng et al., which identified higher BMI and waist circumference as indicators of MAFLD. The early identification of individuals at risk for MAFLD is critical for effective management, and currently, BMI and ultrasonography (FibroScan) are commonly used tools for diagnosis [17]. However, BMI alone is limited, as it cannot differentiate between visceral and subcutaneous fat. Research has shown that other indicators, such as waist circumference, waist-height ratio, body roundness index (BRI), and visceral adiposity index, may offer superior predictive power [18]. Studies by Zheng et al. emphasize the predictive strength of the waist-hip ratio (WHR) for MAFLD. Furthermore, the BRI has emerged as a potentially stronger predictor of MAFLD than BMI, WC, or WHR [17,19].

Visceral fat accumulation, particularly in the liver, is strongly linked to MAFLD and its progression to liver fibrosis, which is often exacerbated by metabolic syndrome (MetS). Albhaisi et al. found that even lean individuals with normal BMI can be susceptible to MAFLD, experiencing more severe hepatic consequences [20,21]. This highlights the complexity of diagnosing MAFLD, particularly in those without overt obesity. Environmental and metabolic factors such as lipodystrophy, metabolic syndrome, hypertension, and insulin resistance are key contributors to MAFLD, especially in lean individuals. All study participants had no history of alcohol consumption, and the etiology of MAFLD is believed to be multifactorial, with a significant genetic component [21,22].

Smoking has been shown to exacerbate MAFLD, with studies including a meta-analysis by Akhavan Rezayat et al. and Liu et al. indicating a strong association between smoking and the development of the disease [23,24]. Our findings align with these studies, showing a significant relationship between smoking history and MAFLD. Additionally, the association between MAFLD and hypertension has been well documented, with Ng et al. suggesting a bidirectional link between these conditions. Our study also found a significant correlation between hypertension and MAFLD, consistent with the findings of Ng et al. MAFLD and type 2 diabetes mellitus (T2DM) are closely linked conditions that often manifest together as components of metabolic syndrome. Research consistently demonstrates that MAFLD significantly elevates the risk of developing T2DM, even in the absence of obesity and other conventional metabolic risk factors. Insulin resistance is a key factor underlying this association, contributing both to the progression of liver disease and to impaired glucose metabolism in diabetic patients [25-27]. Additionally, individuals with MAFLD frequently exhibit other metabolic comorbidities, such as hypertension, a history of tobacco use, and preexisting diabetes, reinforcing the multifactorial nature of metabolic syndrome.

In terms of laboratory findings, raised WBC count has been suggested as an indicator of MAFLD, as it correlates with metabolic syndrome and insulin resistance [28]. Additionally, the MAFLD group exhibited elevated levels of ALT and GGT (385.3 U/L and 105 U/L, respectively) compared to controls (55 U/L and 41 U/L, respectively), supporting the utility of these liver enzymes as markers of hepatic injury [29,30].

In the lipid profile analysis, the MAFLD group had significantly higher levels of total cholesterol, triglycerides, and LDL compared to controls. Notably, the incidence of MAFLD increased with higher total cholesterol and triglyceride levels, while it decreased as HDL levels rose. These findings align with previous studies linking lipid abnormalities with MAFLD [16].

Our study also investigated the role of serum TIMP-2, a natural inhibitor of metalloproteinases involved in the regulation of ECM remodeling. Elevated TIMP-2 levels have been associated with adipose tissue remodeling and metabolic disruptions characteristic of MAFLD. ROC curve analysis revealed significantly higher serum TIMP-2 levels in the MAFLD group compared to controls, with a cutoff value of 8.909 ng/mL achieving a sensitivity of 91% and specificity of 99%. These findings support the potential utility of TIMP-2 as a noninvasive biomarker for MAFLD, in line with earlier research highlighting its role in identifying liver fibrosis.

Furthermore, serum TIMP-2 showed significant correlations with multiple metabolic and biochemical parameters. Positive associations with fasting glucose, HbA1c, and insulin point to its involvement in insulin resistance and impaired glucose metabolism. Strong correlations with liver enzymes and CAP scores suggest a relationship with hepatic fat accumulation and liver injury. Additionally, associations with lipid levels and inflammatory markers imply a link to dyslipidemia and systemic inflammation. Inverse correlations with red blood cell indices, alongside positive associations with renal function markers and electrolytes, reflect broader metabolic disturbances. Collectively, these findings highlight TIMP-2 as a promising marker of metabolic dysfunction, particularly in the setting of MAFLD and type 2 diabetes, further supporting its diagnostic value in clinical practice.

Limitations

The main limitation of this study is the relatively small sample size, which restricts the generalizability of the findings. In addition, the absence of MMP-2 measurements and the inability to calculate the MMP-2/TIMP-2 ratio limit a more comprehensive understanding of the protease-inhibitor balance in disease progression. Furthermore, the lack of liver biopsy validation, considered the gold standard for staging hepatic steatosis and fibrosis, restricts the depth of histopathological correlation. Larger multicenter studies incorporating biopsy data as well as molecular analyses, such as SNP profiling, are warranted to validate and extend these findings. Despite the limitations, this study demonstrates a strong association between serum TIMP-2 levels and MAFLD in an Indian population. It also highlights the link between TIMP-2 and key clinical and biochemical markers related to MetS.

## Conclusions

Our study demonstrates that serum TIMP-2 is a promising marker for diagnosing and monitoring MAFLD, with a cutoff value of 8.909 ng/mL suggestive of MAFLD, showing a sensitivity of 91% and specificity of 99%. It shows a strong positive correlation with both FibroScan values and traditional liver injury markers such as ALT and GGT. Although further research is necessary to validate these findings, the potential of TIMP-2 as a reliable diagnostic tool for MAFLD is evident. Future studies should focus on refining the role of TIMP-2 in MAFLD diagnosis and its application in clinical settings.

## Figures and Tables

**Figure 3:**
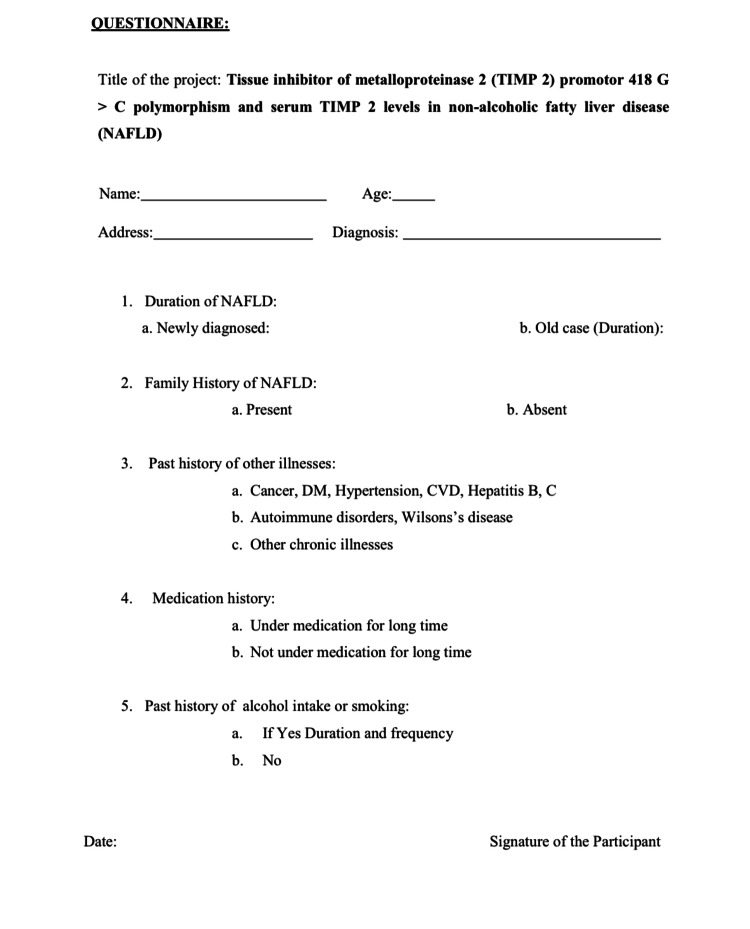
Questionnaire used in this study
